# Anomalous vertebral artery course causing radiculopathy: A case report and review of relevant literature

**DOI:** 10.1016/j.bas.2025.104331

**Published:** 2025-08-06

**Authors:** H. Jabri, K.T. Chen, S. Sharma, Y. Liu, J.L. Kim

**Affiliations:** a Department of Spine Surgery, Prince Sultan Military Medical City, Riyadh, Saudi Arabia; b Department of Neurosurgery, Chang Gung. Memorial Hospital, Chia-Yi, Taiwan; c Orthopaedic Department, Smt. SCL General Hospital, Smt NHL Municipal Medical College, Ahmedabad, India; d Department of Neurosurgery, Seoul St. Mary's Hospital, College of Medicine, The Catholic University of Korea, Seoul, South Korea

**Keywords:** Cervical radiculopathy, Vertebral artery anomaly, Minimally invasive surgery, Ectatic vertebral artery, Surgical management

## Abstract

**Introduction:**

Cervical radiculopathy typically results from degenerative disc disease, but rarely, an ectatic vertebral artery can cause it. This case report and literature review examine a unique instance of cervical radiculopathy due to an anomalous vertebral artery. Vertebral artery loop formation (VALF) is uncommon but can significantly impact surgical outcomes and poses a risk during cervical spine surgeries if unidentified preoperatively.

**Research question:**

To highlight the rare occurrence of cervical radiculopathy caused by an ectatic vertebral artery and discuss its surgical management.

**Material and methods:**

A 73-year-old female with left shoulder pain and C5 nerve root palsy underwent radiological evaluation, revealing an ectatic vertebral artery at C4/5. Treatment involved posterior tubular minimally invasive laminoforaminotomy with microvascular decompression, using intraoperative ultrasound and indocyanine green (ICG) angiography to ensure artery patency.

**Results:**

Post-surgery, the patient showed significant symptom improvement, including increased deltoid power and reduced pain. Radiological follow-up confirmed successful decompression.

**Discussion and conclusion:**

This case emphasizes the importance of recognizing vertebral artery anomalies in cervical radiculopathy cases without apparent disc pathology. Preoperative identification and appropriate surgical planning are crucial to prevent iatrogenic injury. The successful outcome contributes to the limited literature on managing such cases and highlights the effectiveness of minimally invasive surgical approaches combined with advanced imaging techniques. Surgeons should consider anomalous vertebral artery courses as a potential cause of cervical radiculopathy, especially when conventional pathology is absent.

## Highlights

• Vertebral artery loop formation, though rare, can significantly impact surgical outcomes.
• Preoperative identification of vertebral artery anomalies is crucial to prevent iatrogenic injury during cervical spine surgeries.
• Appropriate surgical planning and careful technique are essential when managing these uncommon cases.

## 1 Introduction

The origin of anomalous variation in the vertebral artery (VA) remains unknown but congenital abnormality, or as postulated degenerative theory, might be associated, but its incidence is low. The prevalence of vertebral artery loop formation (VALF) in a cadaveric study of 222 cervical spines was 2.7 % (Curylo et al., 2003). However, in a series of cervicobrachial pain without disc pathology, 13 out of 173 patients had VALF, suggesting that the incidence is around 7.51 % (Paksoy et al., 2003). Vertebral artery course remains one of the rare causes of cervical radiculopathy. Overlooking this anomaly might lead to injury to the vertebral artery during surgery for cervical radiculopathy.

Conventionally, the vertebral artery is divided into four segments (Abd el-et al., 1995). The artery courses dorsally in the first segment after originating from the subclavian artery until it enters the foramen of C6. Within the transverse foramina of C6 to C2 lies the second segment. The distal extracranial segment that is short and tortuous is the third portion which passes through the transverse foramen (TF) of the atlas and then curves backward and medially behind the lateral mass of the atlas, where it then makes a sharp turn to pierce the dura mater, thereby entering the cranium through the foramen magnum. The fourth segment is completely intracranial and ends when the vertebral arteries join at the lower pontine border to form the basilar artery (Caplan et al., 1988; Lang and Kessler, 1991). We shall also discuss our experience and present relevant literature review on the topic.

## 2 Case report

**Fig. 1:**
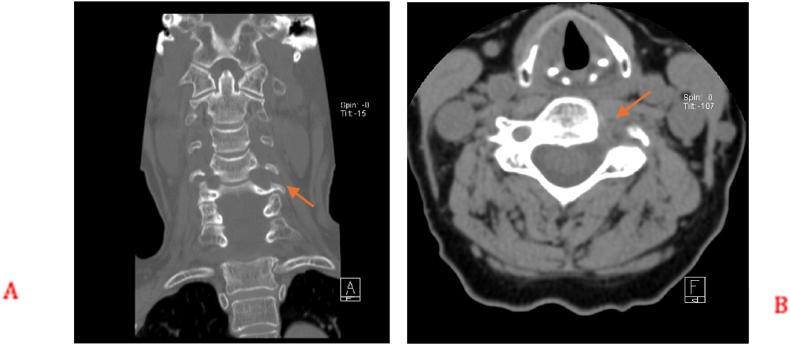
(A) CT spine coronal view showing enlargement of the left C4-5 foramen. (B) Axial CT scan through C4-5 Foramen.

**Fig. 2:**
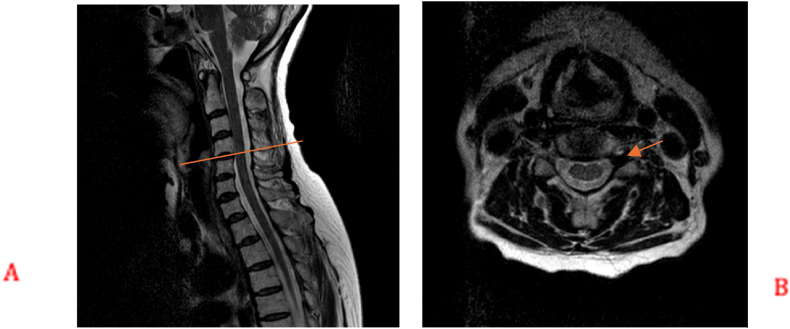
(A) MRI spine sagittal T2 did not reveal overt disc degeneration. (B) MRI spine axial T2 showing abnormal ectatic artery in the left C4-5 foramen.

**Fig. 3:**
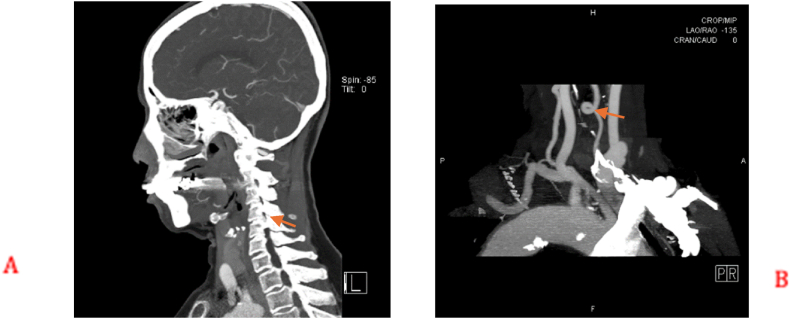
(A) CT angiogram showing the abnormal vertebral artery in the foramen. (B) Tortuosity of the left vertebral artery.

**Fig. 4:**
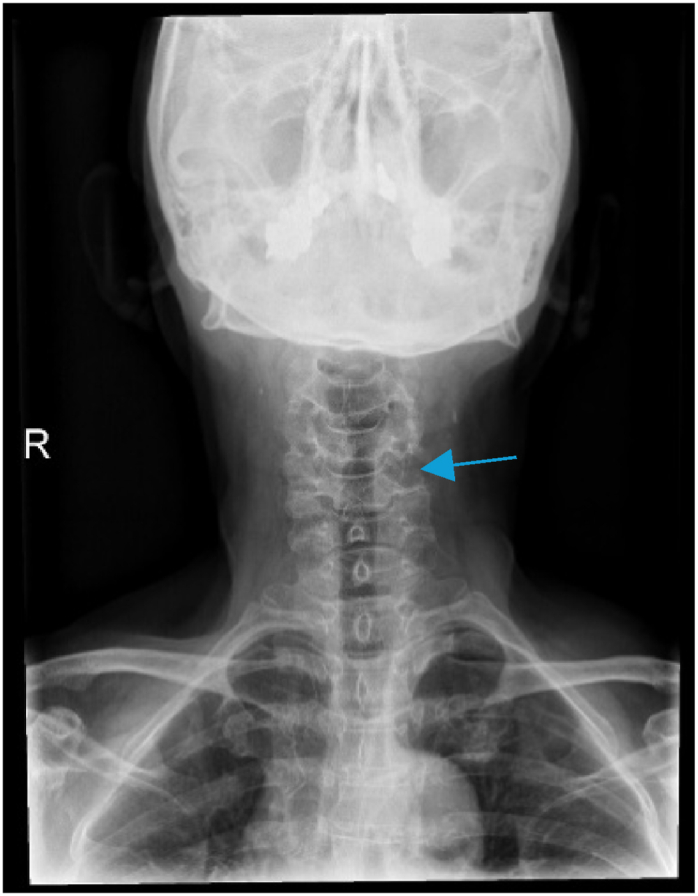
Postoperative X-ray showing the foraminotomy window.

**Fig. 5:**
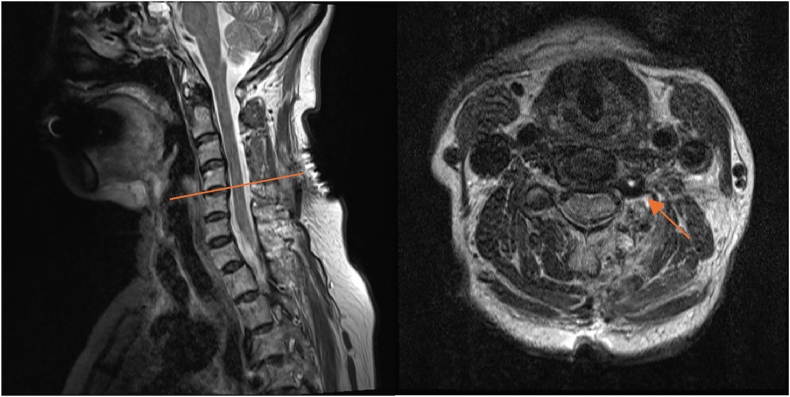
Postoperative MRI sagittal and axial cut showing adequate decompression of the nerve.

A 73-year-old female presented initially with left shoulder pain and brachaligia for which she received conservative treatment measures in another institute for 4 months. Upon arrival to our care, her pain was increasing, associated with left C5 nerve root partial palsy; her deltoid power was 3/5, her visual analog scale (VAS) score was 5 for neck pain and 6 for shoulder pain, while her neck disability index (NDI) was 35.56. Radiologic examination using cervical spine X-ray, computed tomography (CT) scan, magnetic resonance imaging (MRI) and magnetic resonance angiography (MRA), showed enlargement of the left C4/5 intervertebral foramen (Fig. 1). In addition, MRI, MRA, and CT angiogram showed a prominent ectatic vertebral artery in the left C4/5 intervertebral foramen (Fig. 2, Fig. 3), causing compression of the left C5 nerve root, where no associated herniated disc was visible. The radiologic findings were explained to the patient, and a decision was taken to perform surgical microvascular decompression of the nerve root by posterior tubular minimally invasive laminoforaminotomy approach, and interposing Teflon pledget between the artery and nerve. Intraoperative ultrasound and indocyanine green (ICG) were used to confirm the location of the vertebral artery and the patency of the vertebral artery in order to avoid unintentional kinking of the artery at the end of the procedure. The patient had an uneventful postoperative course, and her one-month follow-up showed improved deltoid power to 4/5 with VAS score of 1 for the neck pain and 2 for shoulder pain, while her NDI was 15.56. Postoperative X-ray (Fig. 4) and CT scan showed adequate bony decompression. Finally, MRI further confirmed the complete liberation of the nerve from the offending vessel (Fig. 5).

## 3 Discussion

Cervical radiculopathy is commonly caused by degenerative/discogenic herniated disc. However, other rare causes like ectatic vertebral artery course, congenital lesions, arteriovenous malformation, and tumors should be ruled out, especially when no disc herniation is identified on imaging (Anderson et al., 2014; Kim et al., 2010; Doweidar et al., 2014; Fink et al., 2010). Most surgeons should continue to locate the vertebral artery and its course during the preoperative evaluation for anterior or posterior cervical spine surgery, as this is a crucial step to avoid iatrogenic injury to these vessels (Oga et al., 1996; Peng et al., 2009). As previously mentioned, the prevalence of vertebral artery loop formation (VALF) in a cadaveric study of 222 cervical spines was 2.7 % (Curylo et al., 2003). In another series of cervicobrachial pain without disc pathology, 13 out of 173 patients had VALF, suggesting that the incidence is around 7.51 % (Paksoy et al., 2003). The etiology of this loop formation remains unknown but congenital abnormality, or as postulated degenerative theory, might be implicated. Oga et al., concluded after reviewing 22 cases that the extent of tortuosity is correlated with the severity of cervical spondylosis (Oga et al., 1996). This degenerative theory was also backed by Sakiadia et al., who postulated that the initial step in a process which is flowed by pulsatile pressure leads to migration and erosion of the adjacent structures (Fink et al., 2010). This theory might be supported by the higher occurrence of this pathology in older population.

The delayed symptomatic presentation of this presumably congenital vascular anomaly can be attributed to age-related degenerative changes. We hypothesize that although the arterial tortuosity was present from birth, the cumulative effects of aging - including intervertebral disc height reduction, osteophyte formation, and altered arterial wall compliance - progressively compromised the dimensions of the neural foramen. These physiological changes, absent in earlier decades of life, gradually exacerbated the mechanical impact of the pulsatile tortuous artery until reaching a critical threshold where neurological symptoms became apparent. This temporal relationship between congenital vascular anomalies and delayed symptom onset represents an important consideration in the diagnostic evaluation of patients presenting with similar neurological manifestations later in life (Oga et al., 1996; Sakaida et al., 2009).

Clinical presentation of cases with VALF varies based on pain, radiculopathy, myelopathy or a combination of symptoms; in some instances, vertebrobasilar insufficiency was also reported (Oga et al., 1996). Most patients may be asymptomatic and only diagnosed incidentally during imaging for trauma or other purposes. In a study of 239 symptomatic cervical trauma patients, 6 % of the patients had VALF (Toktaş et al., 2016).

The most common location of VALF in reported cases is C4-5, followed by C3-4, C5-6, and C6-7 and finally C1-2; its usually unilateral with reported cases of bilaterality, mainly in older population typically in the fifth and sixth decades of life (Toktaş et al., 2016). There is a higher prevalence among females rather than males.

In neurologically symptomatic cases, X-ray demonstrates an enlargement of the foramen transversarium with an erosion of the adjacent bone and sclerotic margins related to pulsatile arterial pressure (Sildiroglu et al., 2005). These bony details are better appreciated in axial and reconstructed CT images. Contrasted CT and MRI show signal void in the foramen with nerve compression. Other radiologic examinations such as CT, MRI, and MRA might also be equally informative in demonstrating the course and exact location of tortuosity. Furthermore, Doppler ultrasonography can help intraoperatively in confirming the location of artery (Bruneau et al., 2005). Intraoperative indocyanine green video angiography could be utilized; however, interpretation of the signal intensity should be carefully monitored, which varies with bolus timing, artery distance from the periosteal sheath, and presence of robustious venous plexus (Bruneau, 2010).

These patients' management should start with a trial of conservative management, where some reports confirmed symptom resolution with NSAIDs, blood pressure control, and physiotherapy. Surgery is considered in refractory cases with clear vascular compression or neurological deficit. Surgical decompression can be employed anteriorly, posteriorly, or with combined approaches, as seen in different reports in the literature.

The ultimate surgical goal is freeing the compressed neural structure, whether nerve root or spinal cord, or both. Although, the anterior approach that exposes and frees the vertebral artery carries the risk of recurrent laryngeal nerve and carotid artery injuries. Exposure carried out using laterally passed longus colli to expose the transverse process might damage the sympathetic nerves and may lead to Horner's syndrome. The vessel loop can also be relocated, but the anterior approach is not possible above C3 level.

In the posterior approach, foraminotomy can be performed using minimal invasive surgery tubular dilator or microscopic technique, which indirectly decompresses the nerve, freeing adhesions, with the feasibility of using telfa patch interposed between the nerve and offending vessel loop, hence reducing the likelihood of nerve recompression. Separation of the vessel loop using sling technique(Korinth and Mull, 2007; Tandon et al., 2013) or bypass and reconstruction have been reported in the literature (Sakaida et al., 2009). A technique using a cellular human dermis sling that is wrapped around the VA and sewn to the adjacent paraspinous muscles was reported by Tandon et al. (2013) Blood flow adequacy of the VA should be monitored and evaluated intraoperatively to avoid immediate kinking. One advantage of the sling method is the higher likelihood of separation maintenance. Padding may reduce the pulsatile compression of the offending vessel loop, similar to the surgeries commonly performed in microvascular decompression surgery in cranial pain syndromes.

While bony decompression is the foremost important step in these patients, it might not be adequate to alleviate the patient's symptoms. If either the anterior or posterior approach is adopted, there is a risk of instability, depending mainly on the amount of bone removed to achieve adequate decompression, which could be higher in the posterior approach. Instability in these types of surgery has not been reported in the literature and fusion was not required.

A surgical approach is usually dictated by the extent of every individual patient's pathology, the extent of decompression needed, as well as the surgeon's preference and comfort (Table 1).

**Table 1:** Summary of literature search.

Reference	Symptom	Cervical Level	Approach	Outcome	Comment
**Anderson et al. 1970** (Anderson et al., 2014)	Neck pain	C3-4	Posterolateral foraminotomy	Resolved pain	
**Zimmerman et al. 1970** (Zimmerman and Farrell, 1970)	Occipitocervical neuralgia	C4-5	Posterolateral laminectomy	Resolved pain	
Sharma et al., 1993 (Read More)	Occipital neuralgia	C2-3	Posterolateral laminectomy,VD	Resolved pain	Klippel Feil syndrome, cellulose for VD
**Satoh et al. 1993** (Sharma et al., 1993)	Neck, shoulder, arm pain	C1-2	Suboccipital and C1 decompression	Resolved pain	Suture to anchorvertebral arteries to dura
Duthel et al., 1994 (Umemori et al., 2009)	Cervicobrachial neuralgia, second and third digit paresthesia	C5-C6	Anterolateral, VD	Resolved symptoms	Teflon for VD
**Detwiler et al.1998**(Detwiler et al., 2009)	Cervical and scapular pain	C3-C4	Posterolateral laminectomyand facetectomy, VD	Resolved pain	
**Sakaida et al. 2001** (Sakaida et al., 2009)	C5 radiculopathy	C4-C5	Anterolateral, vascular reconstruction	Resolved pain	End-to-end anastomosis
**Korinth et al. 2007** (Korinth and Mull, 2007)	Neck, shoulder pain	C4-C5	Anterolateral, VD	Resolved pain	Teflon for VD
Dahdaleh et al., 2010 (Dahdaleh et al., 2010)	Presyncope	C2-3, C3-4	Posterior cervical fusion	Resolved symptoms	
**Hage et al. 2012** (H et al., 2012)	Neck pain, C7 radiculopathy	C6-C7	Anterolateral, VD	Resolved pain	
Chibbaro et al., 2012 (Chibbaro et al., 2012)	C6 radiculopathy	C5-C6	Anterolateral, VD	Resolved pain	
Tandon et al., 2013 (Tandon et al., 2013)	Neck, shoulder pain	C4-C5	Anterolateral, VD	Resolved pain	Allograft sling
**Eksi et al. 2016** (Toktaş et al., 2016)	Neck pain, weakness	C5-C6	Posterolateral laminectomy and foraminotomy	Resolved pain	
**Wang et al. 2017** (Duthel et al., 1994)	C6 weakness, paresthesia, radiculopathy	C5-C6	Anterolateral, VD	Resolved symptoms	Teflon for VD
**Wang et al. 2017** (Duthel et al., 1994)	Head, neck pain	C3-C4	Anterolateral, VD	Improved symptoms	
Venteicher et al., 2018 (Venteicher et al., 2018)	C5 weakness, radiculopathy	C4-C5	Anterolateral, VD	Resolved symptoms	Teflon for VD
Yamada et al., 2018 (Yamada et al., 2018)	occcpitalgia, sensory abnormalities	C1-2	Suboccipital craniotomy and C1 laminectomy	Resolved pain	PTEF for VD
**Present study**	Neck, shoulder pain, C5 weakness, radiculopathy	C4-C5	Posterolateral tubular foraminotomy	Improved symptoms	Teflon for VD

## 4 Conclusion

Anomalous origin of the VA may not be the sole reason behind a disease process. However, it may undoubtedly lead to a misdiagnosis during cervical radiculopathy diagnosis. Therefore, detailed information is crucial before any surgery or endovascular intervention in the neck region. Conservative treatment remains the mainstay of management of these anomalies. In refractory cases, surgery is indicated whether approached anteriorly, posteriorly or combined approaches.

The ultimate surgical goal is decompression of the nerve root to alleviate radiculopathy and allow weakness to improve (ICG); by halting the pulsatile compression of the artery on the nerve. Furthermore, the possibility of iatrogenic instability resulting from decompression surgery can be prevented by adding fusion surgery, if needed.

## Declaration of competing interest

The authors declare that they have no known competing financial interests or personal relationships that could have appeared to influence the work reported in this paper.

## References

[bib1] Abd el-Bary T.H., Dujovny M., Ausman J.I. (1995). Microsurgical anatomy of the atlantal part of the vertebral artery. Surg. Neurol..

[bib2] Anderson R.E., Norman Shealy C. (2014). Cervical pedicle erosion and rootlet compression caused by a tortuous vertebral artery. Radiology.

[bib3] Bruneau M. (2010). Video.

[bib4] Bruneau M., Cornelius J.F., George B. (2005). OF the V ERTEBRAL a RTERY to the.

[bib5] Caplan L.R., Vinken P.J., Bruyn G.W., Klawans (1988). Handbook of Clinical Neurology. Vascular Disease. Part 1.

[bib6] Chibbaro S., Mirone G., Yasuda M., Marsella M., Di Emidio P., George B. (2012). Vertebral artery loop - a cause of cervical radiculopathy. World Neurosurg..

[bib7] Curylo L.J., Mason H.C., Bohlman H.H., Yoo J.U. (2003). Tortuous course of the vertebral artery and anterior cervical decompression. Spine.

[bib8] Dahdaleh N.S., Albert G.W., Hasan D.M. (2010). Multiple symptomatic vertebral artery loops treated with posterior cervical fusion. J. Clin. Neurosci..

[bib9] Detwiler P.W., Porter R.W., Harrington T.R., Sonntag V.K.H., Spetzler R.F. (2009). Vascular decompression of a vertebral artery loop producing cervical radiculopathy. J. Neurosurg..

[bib10] Doweidar A., Al-Sayed S., Al-Kandery S. (2014). Symptomatic vertebral artery loop: a case report and review of literature. J. Radiol. Case Rep..

[bib11] Duthel R., Tudor C., Motuo-Fotso M.-J., Brunon J. (1994). Cervical root compression by a loop of the vertebral artery. Neurosurgery.

[bib12] Fink J.R., Leung J.Y., Creutzfeldt C.J. (2010). Vertebral artery loop formation causing severe cervical nerve root compression. Neurology.

[bib13] H Z.A., A S., W D. (2012). Journal of neurosurgery: spineoct 2012/vol. 17/no. 4/pages 337-341ARTICLESurgical management of cervical radiculopathy caused by redundant vertebral artery loop. J Neurosurg Spine J Neurosurg Spine.

[bib14] Kim H.S., Lee J.H., Cheh G., Lee S.H. (2010). Cervical radiculopathy caused by vertebral artery loop formation: a case report and review of the literature. J. Korean Neurosurg. Soc..

[bib15] Korinth M.C., Mull M. (2007). Vertebral artery loop causing cervical radiculopathy. Surg. Neurol..

[bib16] Lang J., Kessler B. (1991). About the suboccipital part of the vertebral artery and the neighboring bone-joint and nerve relationships. Skull Base Surg..

[bib17] Oga M., Yuge I., Terada K., Shimizu A., Sugioka Y. (1996). Tortuosity of the vertebral artery in patients with cervical spondylotic myelopathy: risk factor for the vertebral artery injury during anterior cervical decompression. Spine.

[bib18] Paksoy Y., Levendoglu F.D., Ögün C.Ö., Üstün M.E., Ögün T.C. (2003). Vertebral artery loop formation: a frequent cause of cervicobrachial pain. Spine.

[bib19] Peng C.W., Chou B.T., Bendo J.A., Spivak J.M. (2009). Vertebral artery injury in cervical spine surgery : anatomical considerations , management , and preventive measures. Spine J..

[bib21] Sakaida H., Okada M., Yamamoto A. (2009). Vascular reconstruction of a vertebral artery loop causing cervical radiculopathy and vertebrobasilar insufficiency. J. Neurosurg. Spine.

[bib22] Sharma R.R., Parekh H.C., Prabhu S., Gurusinghe N.T., Bertolis G. (1993). Compression of the C-2 root by a rare anomalous ectatic vertebral artery. J. Neurosurg..

[bib23] Sildiroglu O., Alparslan L., Ganiyusufoglu K., Sirvanci M., Kantarci M. (2005). Cervical radiculopathy caused by vertebral artery loop formation. J. Comput. Assist. Tomogr..

[bib24] Tandon A., Chandela S., Langer D., Sen C. (2013). A novel sling technique for microvascular decompression of a rare anomalous vertebral artery causing cervical radiculopathy. Neurosurg. Focus.

[bib25] Toktaş Z.O., Bayoumi A.B., Yener Y. (2016). Vertebral artery loops in surgical perspective. Eur. Spine J..

[bib26] Umemori T., Kitagawa Y., Sasaki T., Iida T., Satoh S., Yamamoto N. (2009). Cervical cord compression by the anomalous vertebral artery presenting with neuralgic pain. J. Neurosurg..

[bib27] Venteicher A.S., Quddusi A., Coumans J.V. (2018). Anterolateral approach for a cervical nerve root compression syndrome due to an ectatic vertebral artery. Oper. Neurosurg..

[bib28] Yamada Y., Kokubo Y., Kawanami K., Itagaki H., Sato S., Sonoda Y. (2018). A case report: C2 radiculopathy induced by neck flexion due to the cord compression of C2 segmental type vertebral artery relieved by microvascular decompression. Surg. Neurol. Int..

[bib29] Zimmerman H.B., Farrell W.J. (1970). Cervical vertebral vertebral erosion caused vertebral artery tortuosity. Am. J. Roentgenol..

